# Editorial: Chimeric antigen receptor T cell therapies and bispecific antibodies in hematologic malignancies

**DOI:** 10.3389/fonc.2026.1936446

**Published:** 2026-08-18

**Authors:** Yangmin Zhu

**Affiliations:** The Affiliated Guangdong Second Provincial General Hospital of Jinan University, Guangzhou, Guangdong, China

**Keywords:** bispecific antibodies, chimeric antigen receptor, hematologic malignancies, leukemia, lymphoma, myeloma

The past decade has witnessed a paradigm shift in the treatment of hematologic malignancies, driven by the emergence of T-cell redirecting immunotherapy. Chimeric antigen receptor T-cell (CAR-T) therapy and bispecific antibodies (BsAbs) have revolutionized the outcomes of patients with relapsed or refractory B-cell lymphomas, leukemias, and multiple myeloma. However, the rapid clinical adoption of these sophisticated modalities has outpaced our ability to definitively compare them, select optimal candidates, and manage their distinctive toxicity profiles. This Research Topic assembles original research, systematic reviews, and clinical case reports that collectively illuminate the current state of these therapies and chart a course for their optimal integration into clinical practice.

The comparative effectiveness of CAR-T and BsAbs represents a central clinical question. He et al. provided the largest meta-analysis addressing this in relapsed/refractory follicular lymphoma, demonstrating superior complete response rates (82% vs. 65%) and progression-free survival with CAR-T therapy, although at the cost of increased neurotoxicity. Multivariate analysis revealed that CAR-T was not an independent predictor of response after adjusting for confounders, suggesting that patient- and disease-specific factors, including tumor burden, prior treatment refractoriness, and disease stage, significantly influenced outcomes. This nuanced finding challenges the notion of universal superiority and emphasizes the importance of individualized treatment.

Expanding this comparison to the broader B-cell non-Hodgkin lymphoma population, Wang et al. synthesized data from 59 trials and 2,914 patients. Their benefit-risk analysis elegantly positioned CD19/CD22 dual-targeted CAR-T in the most favorable efficacy-safety zone, while CD3×CD20 BsAbs occupied a region characterized by lower efficacy but superior safety. This quantitative framework provides clinicians with actionable guidance: CAR-T is preferred when deep, durable remission is the priority, whereas BsAbs are better suited for frail patients, those with limited access to specialized centers, and individuals who cannot tolerate the logistical demands of cellular therapy.

The clinical application of these therapies in real-world populations, often excluded from pivotal trials, has been explored through several instructive case reports. Ibraheem et al. demonstrate that CAR-T can be safely delivered to patients with pre-existing Parkinson’s disease through multidisciplinary optimization, including modified neurotoxicity monitoring, tailored dopaminergic support, and deliberate selection of a 4-1BB-based construct with lower neurotoxicity risk. This case exemplifies how thoughtful product selection and proactive supportive care can extend the benefits of CAR-T therapy to vulnerable populations. Liu et al. report durable complete remission in refractory plasmablastic lymphoma using sequential CD19 and CD22 CAR-T, illustrating the potential of dual-targeting to circumvent antigen escape—a major resistance mechanism that limits single-target approaches.

Toxicity management is a critical aspect of these therapies. Akiyama et al. presented the first case of an autologous stem cell boost successfully rescuing severe cytopenia induced by BCMA-directed bispecific antibody therapy in multiple myeloma, extending this salvage strategy beyond the CAR-T setting. This report highlights the importance of early stem cell collection, particularly as patients receive increasingly intensive T-cell-engaging therapies that may compromise hematopoietic reserves. Abuhelwa et al. characterized post-progression patterns after BCMA-directed CAR-T in myeloma, demonstrating that extramedullary disease at baseline or progression confers significantly inferior survival. These findings underscore the aggressive nature of extramedullary disease and the urgent need for targeted bridging and maintenance strategies in this high-risk subgroup of patients.

The sequential application of these modalities represents an emerging area of research. Oura et al. report the first case of epcoritamab efficacy in neurolymphomatosis, achieving complete remission after ibrutinib failure. The authors propose that BsAbs may serve as a valuable bridging strategy for patients with impaired performance status, allowing functional recovery while awaiting CAR- T cell manufacturing. This concept of sequential therapy—using BsAbs to stabilize the disease and restore physical function before proceeding to definitive cellular therapy—represents a practical approach to expanding treatment access. Chen et al. extend the BsAb application to light chain amyloidosis, demonstrating that teclistamab can induce rapid hematologic complete remission, though renal function improvement remained limited—a reminder of the organ-specific challenges in this disease.

Diagnostic precision is equally important to therapeutic efficacy. Ibraheem and Dalby addressed the critical need to distinguish cytokine release syndrome from sepsis, a clinical dilemma that frequently complicates CAR-T management. The DRACARYS study protocol proposes the IL-6/procalcitonin ratio as a bedside biomarker to guide appropriate interventions. This study exemplifies the evolution from efficacy-focused clinical trials to supportive care optimization, recognizing that toxicity management is integral to therapeutic success and that accurate diagnosis prevents both unnecessary immunosuppression and inappropriate antibiotic exposure.

A comprehensive review by Amirmokhtari and Lutfi synthesizes the pivotal trials that led to FDA approvals for hematologic malignancies while examining resistance mechanisms and next-generation constructs. The long-term follow-up from CARTITUDE-1, demonstrating that one-third of multiple myeloma patients remain progression-free at five years, provides compelling evidence that CAR-T may offer curative potential in this previously incurable disease. The review also critically examines novel approaches—armored CAR-T, *in vivo* CAR-T, allogeneic CAR-T, and dual-targeting strategies—that aim to overcome current limitations, including T-cell exhaustion, antigen escape, and logistical barriers.

Collectively, this Research Topic establishes that CAR-T and BsAbs are not competing therapies but rather complementary tools within an evolving therapeutic armamentarium. The optimal strategy requires an individualized risk-benefit assessment considering patient fitness, disease kinetics, treatment urgency, and prior therapy exposure. CAR-T offers superior efficacy at the cost of greater toxicity and logistical complexity, whereas BsAbs provide accessible, off-the-shelf options with favorable safety profiles but potentially less durable responses. Future research should prioritize head-to-head randomized trials with biomarker stratification, optimized sequencing strategies, and toxicity mitigation in high-risk populations. As these immunotherapies expand beyond hematology into solid tumors and autoimmune diseases, the lessons learned from this Research Topic will inform broader applications, ultimately improving outcomes across the spectrum of immune-mediated diseases. The integration of these powerful modalities into thoughtful and personalized treatment pathways represents the next frontier in cancer immunotherapy.

